# Secondary or Recurrent Hemorrhage*A paper read Oct. 2, 1882, before the Buffalo Medical and Surgical Association.

**Published:** 1882-11

**Authors:** Charles C. F. Gay

**Affiliations:** Surgeon to the Buffalo General Hospital


### An Inquiry into the Source and Cause of Secondary or Recurrent Hemorrhage, after Ligation of Arteries for Aneurism.*

By Charles C. F. Gay, M. D.

Surgeon to the Buffalo General Hospital.

The subject to which your attention, this evening, is invited, is an important one. I cannot conceive of any subject which is invested with greater surgical interest than an "Inquiry into the source and cause of secondary or recurrent hemorrhage after ligation of arteries for aneurism." I am sure no one could have failed to observe the numerous reports of cases of aneurism which are scattered all through the pages of our home and foreign medical journals. There is one special fact connected with aneurism which gives to it peculiar interest and importance, without which the subject would be divested of much of its value; it is the fact of the liability, and, indeed, certainty of the supervention of secondary hemorrhage after ligation, by which, without warning, life is put in jeopardy. Lister has labored for two full years to prepare and perfect the so-called chromic cat-gut ligature. Richard Barwell has written and published a book, the chief aim and object of which seems to have been to describe the preparation and efficiency of his ox-aortic flat

*A paper read Oct. 2, 1882, before the Buffalo Medical and Surgical Association.

ligature. By the employment of these ligatures, or either one of them, these gentlemen hope to prevent secondary hemorrhage after ligation of arteries.

It is quite patent, therefore, that a subject which so largely engrosses the time of such men as these, possesses very great surgical interest and importance and is altogether worthy of your attention and study.

If one has had much occasion to witness hemorrhages following ligation for aneurism, and has bestowed some considerable thought upon its origin, he will be led to ask: Whence originates the hemorrhage? Whence its source and what its cause?

Let us briefly interrogate some of our authors, and see what responses may be obtained:

"I think that the presence of a collateral branch in close proximity to the *distal* side of the ligature, more especially if it be one that serves to carry on the anastomosing circulation, will be found to have a decided tendency to prevent the occlusion of the distal end of the artery, and thus to favor the occurrence of secondary hemorrhage."—ERICHSEN.

"The bleeding often supervenes without any assignable cause, generally suddenly and unexpectedly. * * * When proceeding from a large vessel it may prove fatal in a few minutes, in the same manner as when the bleeding is primary. The scarlet hue of the blood always denotes its source."—GROSS.

"The sloughing condition of the soft parts, and an imperfect thrombus, are concerned in determining the hemorrhage, * * * and these depend upon a defective state of the blood and an imperfect nutrition of the tissues about the wound."—AGNEW.

"From ulceration and sloughing of an artery spreading through the arterial tunics, imperfect closure of arteries, when a ligature separates through the influence of a diseased state of the artery."—DRUETT.

"Excessive accumulation of blood determines rupture of the capillaries."—ROKITANSKY.

Guthrie and Porter affirm that the blood in the great majority of instances comes from the distal, and not from the proximal side of the wound.

Lister and Barwell believe secondary hemorrhage is entirely owing to the character and application of the ligature.

Authors are all in accord in assigning one or more of the following circumstances as conditions precedent to the occurrence of secondary hemorrhage.

1st. Faulty structure of the ligature.

2d. Imperfect application of the ligature.

3d. Diseased state of the coats of the artery.

4th. Irritability of the arterial system.

It is my purpose to make inquiry into some of these conditions, without, however, following the order in which they are herein named—but rather to consider them grouped together—and to add thereto some remarks upon cases, the history and course of which I have clinically observed and noted. Since Barwell and Lister attribute hemorrhage to the imperfect application and faulty construction of the ligatures, it will not be regarded, I trust, an unwarrantable digression, should I say something concerning these ligatures.

Barwell's flat ligature is prepared from the aorta of the ox in a fresh condition and placed in a three per cent. solution of carbolic acid. It is believed by the author when thus prepared to be hemorrhage-proof. He says: "To make out of the aorta a ligature, it is only necessary to peel away the outer cellular coats, and then, with a pair of scissors, skillfully to cut the middle and outer coats spirally round and round, taking care to keep the breadth equable." This flat ligature does not divide the arterial coats, but leaves the vessel entire, secure he says, therefore, from secondary hemorrhage. "By its use no excuse or occasion for secondary hemorrhage is given." Thus we recognize the above statement as the expression of a belief that hemorrhage is caused wholly and alone by rupture of the arterial tunics. My present impression is that Barwell has not yet reported a sufficient number of successful cases fully to establish the claim, for his ligature, of infallibility.

Lister's chromic cat-gut ligature—which is really not cat-gut at all, but is made, as all such ligatures are, from the small intestines of the sheep, for an account of the preparation of which I refer you to the *London Lancet* for April, 1881, p. 296—is much extolled by Lister. By its use Lister hoped to eliminate all risk of secondary hemorrhage. It is so well prepared that the ligation of arteries may be done under aseptic and antiseptic precautions. But I have noticed that this ligature failed in the hands of Bartleet, who reports a case to the British Medical Association at its recent meeting, an account of which may be found published in the New York *Medical Record* for Sept. 9, 1882. The wound, after ligating the common femoral trunk, did not remain aseptic and secondary hemorrhage came on. Still later, Sept. 23d, another failure of the cat-gut ligature is reported. Autopsy made the startling revelation that the sutures were untied. Quite recently I ligated the superficial femoral artery for aneurism, applied the tourniquet and instructed my patient how to turn the screw in case there should be any bleeding. On the ninth day, suddenly, there was a profuse hemorrhage, and by the time the patient had tightened the tourniquet, the bed clothes and blanched appearance of the patient's face denoted much loss of blood.

Getting to the patient as soon as possible, I laid open the wound, sponged it out, lifted up the artery by making traction with the ligature, and made a thorough and careful examination of the artery and its surroundings. The artery showed a vermilion border or ring upon either side the ligature; it was firmly tied, indeed, so little change was manifest since the ligature was applied that one could not avoid pronouncing its application faultless, therefore it was not re-inforced by another.

There was no further hemorrhage after removal of the tourniquet. Fruitless search was made for the source of the hemorrhage. I had good opportunity thoroughly to examine the wound and the ligated artery. There was no appearance upon the distal, and none, of course, upon the proximal side of the ligature, of inflammatory softening or fatty degeneration of tissue nor atrophy. The arterial tissue showed great resistance to destructive elements. The evidence which I expected to find of faulty ligation, rupture of the arterial tunics or softening, was absent, hence the conclusion, that the source of hemorrhage must be sought for elsewhere or otherwise explained. The wound was dressed by placing over it a compress and bandage. There was no subsequent hemorrhage. I am unable to say whether it ceased spontaneously or from pressure of the instrument, but am inclined to believe it was the former, since there was no recurrence of it, when pressure was removed. The ligature came away on the twenty-first day. This by no means is a phenomenal, but rather a representative case. I have notes of others, one only of which I will take time to notice. It was a case in which secondary hemorrhage occurred nine days after ligature was placed around the subclavian artery for axillary aneurism. The aneurismal sac had burst, immediately after which I tore the sac freely open so as to enable the hand to be introduced for the purpose of compressing the axillary artery. I then proceeded to ligate the artery. There were, consequently, two wounds: one made by ligating the artery, the other by rupture of the sac. Hemorrhage occurred in the latter wound and was arrested, presumably, by plugging and compress, but possibly the arrest was spontaneous.

This, like the former case, recovered without further ligation. The ligatures, employed in both cases, were made of silken thread; they were large and rendered aseptic by being soaked in a solution of carbolic acid. It is a noteworthy fact that the hemorrhage did not occur in the wound where the ligature was applied, but in the wound remote from it, made by rupture of the sac. I cannot attempt any explanation of this.

The same degenerative process which affects arterial tissues also implicates the veins in close proximity, and as the latter are less resistant to destructive, or degenerative processes, it would not be a far-fetched conclusion to hold them accountable for the hemorrhage; and since it is shown that hemorrhage is suppressed by compress without re-deligation, we are nearly or quite able to reach the conclusion that the veins and not the arteries may be held accountable in some of these cases.

I think I do not expose myself to adverse criticism by affirming that secondary hemorrhage most often occurs on or about the ninth day. Is not this a fact of some significance? An answer to this query may throw some light upon our subject and assist in some measure to resolve our doubts. The pathologist, Rokistansky, above quoted, states that excessive accumulation of blood determines rupture of the capillaries. There must needs be excessive accumulation of blood at one place and the want of it at another, after deligation of an artery, in continuity. The condition of the parts in close proximity to the ligated vessel may be likened to an obstructed water course. Hydrostatic pressure—from a large accumulation of fluid—exerted for a long time in one direction, forces at length a breach, and the obstruction is overcome.

The overflow at first, like a flood, soon subsides, and the stream returns to its normal channel. After ligation in continuity, the blood is dammed up; the circulation is obstructed; the relation of the vessels and their contents is interrupted but not destroyed; the limb does not perish because nourished by the lesser circulation carried on by the anastomotic or collateral vessels, until the larger and normal circulation, which requires a much longer time than the former, has become fully established. Now the capillaries, or the veins, or the degenerate artery upon the distal side gives way and no longer acts as a barrier against the onset of a profuse hemorrhage. The collateral or temporary circulation may require no more than a few hours to form, but the normal and permanent circulation requires several days, it may be, for its establishment, and when the transposition occurs, the danger from hemorrhage arises. It is a law of nature, applicable to all fluid bodies, that they seek, and endeavor to maintain, their natural channels; if turned aside, for a time, by artificial means, they surely again return to the channels designed for their onward, steady flow.

The tendency of the blood current is, I believe, at first, to pass through arterial anastomoses rather than toward the capillaries. In St. Bartholomew's Hospital Reports, I find the following experiment, reported by William Harrison Cripps. He says: "If water be injected into the common iliac artery of a rabbit it will circulate through the capillaries, returning by the vein. If the external iliac and the superficial femoral be now tied, the water will still return by the vein, only much more slowly. If the femoral artery be now divided below the ligature and water still injected by the common iliac, it will be found that a greater quantity of the water will return through the cut (lower) end of the femoral than by the iliac vein." This experiment shows, at least in the case of the rabbit, that even with the iliac vein open, less resistance is afforded by the anastomoses of the arteries than is afforded by the capillaries. This writer has collected fifty-three cases from various sources and classified them according to the plan of treatment. The result of treatment is thus shown:

| | Died. | Recoveries. |
|---|---|---|
| Ligation of external iliac, - - | 12 | 2 |
| Amputation, - - - | 2 | 3 |
| Re-opening wound, - - | 7 | 5 |
| Pressure and bandaging, - | 3 | 12 |
| No treatment, - - - | 4 | 0 |
| Total, - - | | 53 |

The percentage of recoveries, as here shown, is greatly in favor of suppressing hemorrhage by pressure and bandaging. Religation, being found unnecessary to control hemorrhage, constitutes in itself, I think, a partial concession to the theory that the hemorrhage is venous in these cases, and not arterial; or, if not venous, then *arterial from rupture of a branch of the*

*anastomotic system of vessels, from which the blood derives its scarlet hue.*

Unhappily, autopsies throw but little light upon our subject. Barwell reports one. The case was where death followed in two months after the ligation of the carotid artery, but as no secondary hemorrhage is reported during its treatment, it possesses only negative value in this connection, and is now only referred to here to show the condition of the vessels which have been cut through by the ligature. He says: "The artery remained open below the seat of ligatures to within half an inch of its origin, where it was sealed by a plug of coagulum. Above, the vessel was likewise patent as far as the bifurcation, where it was closed by the coagulum, which filled the contracted aneurismal sac. The portion of artery below the ligature contained pus, mixed with what appeared to be broken-down clot, while the part above the ligature was empty. The vein was closed at a point even with the aneurism by a thin plug of bright, golden-colored lymph, while it was plugged below by coagulum." As there is no report of secondary hemorrhage, the conclusion is that we need not always expect it to occur at the place of ligation after or even before the artery is divided.

The natural and legitimate inference, to be drawn from the foregoing, is that our knowledge concerning the source and cause of secondary hemorrhage is at present in a transitional state. One fact, however, is well established and settled: We have demonstrable evidence that the bleeding does not always occur at the place where the ligature is applied. In the main, when we attempt to search for the source and cause of secondary hemorrhage, we grope our way in the dark; we have not sufficient light; we are left in doubt, and labor under difficulties like unto those who make fruitless explorations in search of the source of the Nile. Authors are less full and explicit in their discussion of this theme than we could wish. Says Gross: "The bleeding supervenes often without any assignable cause." The subject is open for discussion, and invites further research.

Its practical bearings ought not to be lost sight of, since prompt and proper treatment—it may be—by plugging and bandaging, may surely save life, while religation nearer the trunk might as surely destroy it. The efficacy of treatment, therefore, depends upon the knowledge we have at our instant command of the true source and cause of all hemorrhages after deligation of arteries for aneurism.

